# Upgrading disclosure statements for speakers and authors on nephrology: involvement in direct patient care

**DOI:** 10.1007/s40620-025-02266-w

**Published:** 2025-03-18

**Authors:** Jadranka Buturović Ponikvar, Giorgina Barbara Piccoli

**Affiliations:** 1 Department of Nephrology, University Medical Centre Ljubljana, Ljubljana, Slovenia; 2 Department of Medicine, Department of Medical Ethics, Faculty of Medicine, University of Ljubljana, Ljubljana, Slovenia; 3 Centre Hospitalier Le Mans, Le Mans, France

Traditionally, a disclosure statement includes detailed information about the financial relationship each author of an article has with industry [1]. However, no information is available on whether a speaker or author on a clinically relevant topic is involved in direct patient care. This information would allow the audience to know whether the author is presenting data and views solely from literature, or indirect experience, or if they balance theoretical knowledge with real-life experience in caring for patients. Disclosing involvement in direct patient care by speakers and authors would improve transparency, foster greater respect and trust between speakers and their audiences, and focus attention on the ever-central role of clinical experience.

Recently, a commentary by Dugdale in *Kidney International Reports*, titled “Paradigm Shift: Dialysis as a Terminal Condition” suggested that nephrologists should initiate “death talks” with dialysis patients [2]. The author commented on the original study by Russwurm et al. [3] published in the same issue. In the article, Dugdale referenced her book on the art of dying, noting that the phrase “the art of dying” (*ars moriendi*) originated in the Middle Ages during the bubonic plague pandemic.

Labeling a health condition as terminal can have serious consequences. It could be understood to mean that millions of maintenance dialysis patients worldwide are suffering in vain, and it is pointless for them to continue to live. As experienced (in other words older) clinicians reading this commentary, we wondered whether the author, affiliated with her hospital's Clinical Ethics Department, had ever been involved in the daily clinical care of dialysis patients, or participated in the long, and sometimes beautiful journeys undertaken by patients on long-term dialysis [4].

Initiating “a death talk”, a highly sensitive and deeply personal issue, may not be universally beneficial for patients and physicians, or for fostering a trusting patient-physician relationship. The relationship people have with death is highly culturally sensitive. Psychologists have shown that people often fail to accurately predict their future feelings and decisions about the end of life [5]. Advance care planning has not yielded the expected results [6]. Additionally, engaging in “a death talk” requires finding the right time and place. Busy physicians working in assembly-line environments, subject to productivity metrics that can lead to burnout and emotional distress, have limited time and are seldom trained to discuss psychologically sensitive issues. Maintenance dialysis patients are usually dialyzed with other patients present, limiting privacy. After hemodialysis, they hurry home to live their lives. Would systematically asking them to discuss their death make them feel better?

**Fig. 1:**
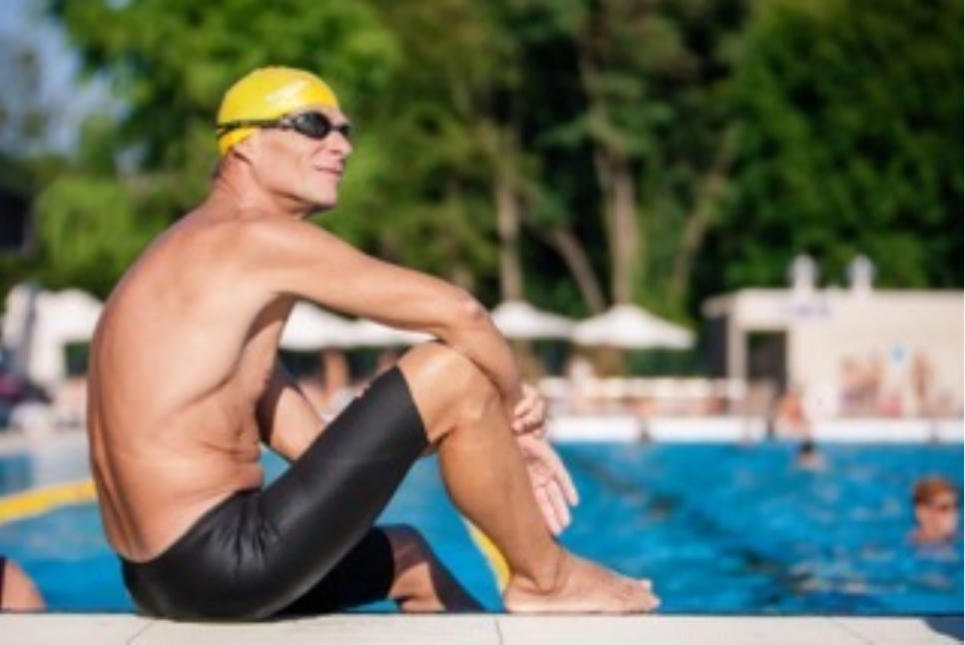
Marathon swimmer, maintenance hemodialysis patient for almost 2 decades, competing (and winning) with (healthy) swimmers in his age group

Maintenance dialysis patients, far from being terminal, can be viewed as examples of the *art of living*. This is how we have seen them over decades of working, living and growing older alongside them. It is not surprising that psychologists have shown interest in this unique patient group [7]. These patients, living on the edge between life and death are often still enjoying a full life, and can teach us a great deal. Each hemodialysis procedure prolongs a patient's life for a few days, until their next session. No one forces them to continue dialysis; they can stop whenever they decide. Yet withdrawals from treatment are rare in Southern and Eastern Europe. Our patients are usually aware that their life depends on well-functioning yet fragile systems. Natural disasters, pandemics, and wars put dialysis patients at immediate risk of death. Initiating an unsolicited “death talk” with such patients could be cruel. It could induce the fear that something is changing in their clinical condition, or even that their access to dialysis might be restricted (Fig. 1).

While fully acknowledging the importance of ethical and philosophical issues in enriching discussions on clinical approaches, in cases like the one mentioned here, a disclosure statement about the author's involvement in direct patient care in dialysis would have added value by putting the thesis discussed in this editorial in context. The opinions and interpretations of physicians directly involved in kidney care may differ, be more “experience-based”, and possibly more partisan, than those of physicians analyzing the situation from a distance. Lack of direct involvement may favor intellectual independence, and offer new viewpoints. Both are needed, and should ideally be combined.

Disclosing involvement in direct patient care might be important also when drafting guidelines. Guidelines, whose aim is to provide detailed and extensive support for clinical decisions, are the products of evidence-based medicine. However, the way they are implemented is critical and a note at the beginning or end of a talk or text [1], usually emphasizes that the guidelines are not meant to determine treatment of individual patients nor represent the standard of care. Unfortunately, this information is often neglected and compliance with guidelines is often monitored as part of quality assessment. Each patient is unique, however, safeguarding a specific patient is determined by the specific decisions of their physician. By acknowledging the current and past clinical involvement of the guideline, authors may once again help contextualize them, thereby leading to an appreciation of the “case mix” of clinical, epidemiological, and ethical expertise involved.

Such a “clinical” disclosure is also likely to encourage the inclusion of more physicians deeply involved in direct patient care as co-authors of guidelines. These physicians may publish less, but may better understand the risks and benefits, and the barriers that need to be overcome in clinical practice. This would complement the presence of patients and of other members of the healthcare team that are now increasingly involved in guideline production.

A proposal for a structured disclosure report on involvement in direct patient care is presented in Table 1.

**Table 1:**
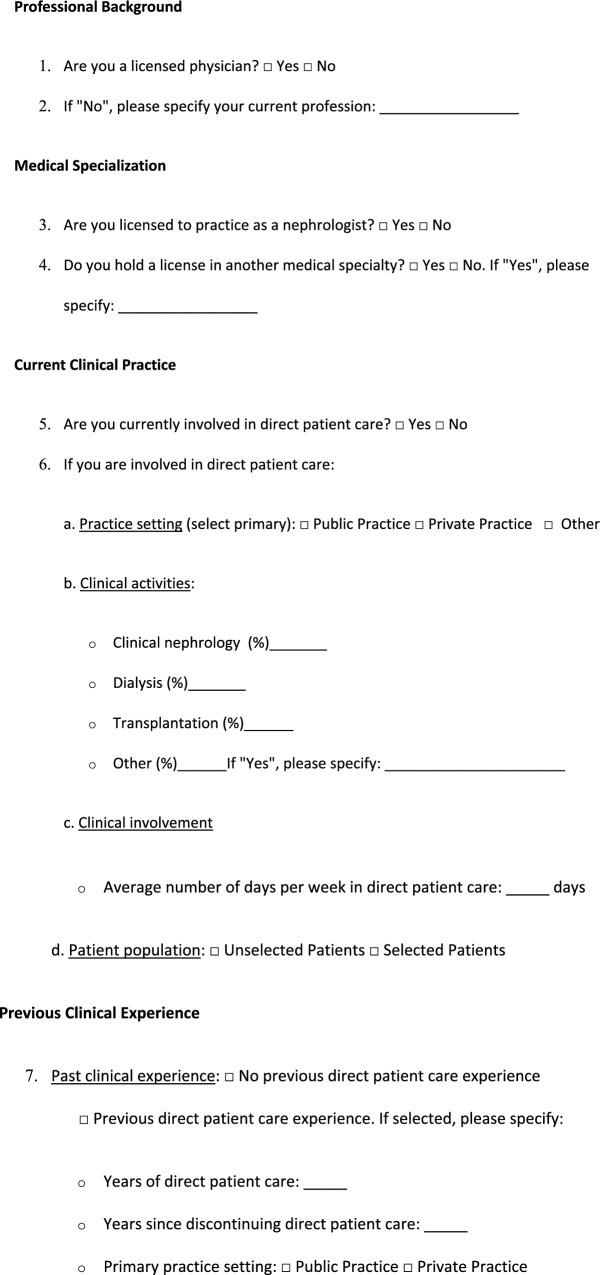
Proposal for upgrading disclosure—involvement in direct patient care

Practicing nephrologists constantly deal with medical and ethical decisions; the decision-making process is becoming increasingly complex and many “strangers at the bedside” [8] (now including even artificial intelligence) are involved in what is called “shared decision-making”. However, accountability for any harm caused will primarily remain with the physician. This is unlikely to change any time soon, if ever, and should probably never change; while “strangers at the bedside” may be welcome, medical time needs to be better acknowledged to enable us to provide timely, safe, and high-quality care. Our proposal to upgrade disclosure statements in Nephrology could be a step in the direction of acting in the best interest of our patients, and of defending the importance of dedicated time physicians can spend with them [9]; it is an investment in the future, not only of medicine, but also of each of us; after all, sooner or later we will all be patients.
